# Dexamethasone can attenuate the pulmonary inflammatory response via regulation of the lncH19/miR-324-3p cascade

**DOI:** 10.1186/s12950-020-00266-0

**Published:** 2021-01-07

**Authors:** Ye Chen, Chao Zhang, Chang-xue Xiao, Xiao-dong Li, Zhi-li Hu, Shou-di He, Xiao-jun Xiao, Feng Xu

**Affiliations:** 1 Department of Pediatric, Huazhong University of Science and Technology Union Shenzhen Hospital, Shenzhen, 518052 China; 2 Department of Pediatric Intensive Care Unit, Children’s Hospital of Chongqing Medical University, No. 136 Zhongshan two road Yuzhong district, Chongqing, 400013 China; 3 Ministry of Education Key Laboratory of Child Development and Disorders, Children’s Hospital of Chongqing Medical University, Chongqing, 400013 China; 4 National Clinical Research Center for Child Health and Disorders, Children’s Hospital of Chongqing Medical University, Chongqing, 400013 China; 5 China International Science and Technology Cooperation base of Child development and Critical Disorders, Children’s Hospital of Chongqing Medical University, Chongqing, 400013 China; 6 Chongqing Key Laboratory of Pediatrics, Children’s Hospital of Chongqing Medical University, Chongqing, 400013 China; 7 Department of Neonatology, Huazhong University of Science and Technology Union Shenzhen Hospital, Shenzhen, 518052 China; 8 Traditional Chinese Medicine Department of Rheumatism, Huazhong University of Science and Technology Union Shenzhen Hospital, Shenzhen, 518052 China; 9 Department of Medicine, Research Center of Allergy & Immunology, Shenzhen University, Shenzhen, 518055 China

**Keywords:** Pulmonary, Inflammatory response, Dexamethasone, lncRNA, miRNA

## Abstract

**Objective:**

To investigate lncRNAs and their roles in regulating the pulmonary inflammatory response under dexamethasone (Dex) treatment.

**Methods:**

IL-1β (10 ng/mL) and LPS (1 μg/mL) was used to construct inflammatory cell models with A549 cells; IL-1β performed better against LPS. Different concentrations of Dex were used to attenuate the inflammation induced by IL-1β, and its effect was assessed via RT-PCR to detect inflammatory cytokine-related mRNA levels, including those of IKβ-α, IKKβ, IL-6, IL-8, and TNF-α. Furthermore, ELISA was used to detect the levels of the inflammatory cytokines TNF-α, IL-6, and IL-8. RT-PCR was used to quantify the levels of lncRNAs, including lncMALAT1, lncHotair, lncH19, and lncNeat1. LncH19 was most closely associated with the inflammatory response, which was induced by IL-1β and attenuated by Dex. Among the lncRNAs, the level of lncH19 showed the highest increase following treatment with 1 and 10 μM Dex. Therefore, lncH19 was selected for further functional studies. LncH19 expression was inhibited by shRNA transduced with lentivirus. Cell assays for cell proliferation and apoptosis as well as RT-PCR, western blot, and ELISA for inflammatory genes were conducted to confirm the functions of lncH19. The predicted target miRNAs of lncH19 were hsa-miR-346, hsa-miR-324-3p, hsa-miR-18a-3p, hsa-miR-18b-5p, hsa-miR-146b-3p, hsa-miR-19b-3p, and hsa-miR-19a-3p. Following estimation via RT-PCR, hsa-miR-346, hsa-miR-18a-3p, and hsa-miR-324-3p showed consistent patterns in A549 NC and A549 shlncH19. An miRNA inhibitor was transfected into A549 NC and A549 shlncH19 cells, and the expression levels were determined via RT-PCR. hsa-miR-324-3p was inhibited the most compared with hsa-miR-346 and hsa-miR-18a-3p and was subjected to further functional studies. RT-PCR, ELISA, and western blotting for inflammatory gene detection were conducted to validate the functions of the target hsa-miR-324-3p.

**Results:**

Treatment with 1 and 10 μM Dex could effectively attenuate the inflammatory response. During this process, lncH19 expression significantly increased (*P* < 0.05). Therefore, treatment with 1 μM Dex was used for further study. Under IL-1β treatment with or without Dex, lncH19 inhibition led to an increase in cell proliferation; a decrease in cell apoptosis; an increase in the protein levels of inflammatory genes; phosphorylation of P65, ICAM-1, and VCAM-1; and increase inflammatory cytokines. Prediction of the targets of lncH19 and validation via RT-PCR revealed that miR-346, miR-18a-3p, and miR-324-3p negatively correlate with lncH19. Additionally, Dex increased the lncH19 expression but reduced that of the miRNAs. Among the miRNAs, miR-324-3p was the most markedly downregulated miRNA following treatment of miRNA inhibitors. The MTS assay and cell apoptosis assay showed that the miR-324-3p inhibitor inhibited cell proliferation and induced cell apoptosis, thereby significantly attenuating the inflammatory response, which reversed the effect of lncH19 in regulating cell proliferation and the secretion of inflammatory cytokines (*P* < 0.05). Therefore, lncH19 might regulate miR-324-3p in pulmonary inflammatory response under Dex treatment.

**Conclusion:**

Dex can attenuate the pulmonary inflammatory response by regulating the lncH19/miR-324-3p cascade.

## Introduction

Pulmonary inflammatory defense is initiating by mobilizing inflammatory cells and phlogistic factors into the lung [1]. Airway epithelial cells activation is the first line of defense for airway and lung from inflammation [2]. Epithelial cells recognize microorganisms via toll-like receptors; activate the NF-κΒ signaling pathway; and increase airway epithelial inflammatory cytokines, such as IL-8, IL-1α, and tumor necrosis factor-α (TNF-α) [3]. However, immune imbalance caused by excessive or chronic inflammation can lead to a variety of lung diseases, including asthma, acute lung injury and diffuse interstitial disease [4]. Inflammatory cytokines are involved in inducing, prolonging, and amplifying the inflammatory response; inducing airway hyperresponsiveness by promoting the growth, proliferation and differentiation of eosinophils, and promoting the generation of allergy-specific immunoglobulin E. Wilson. found that some of the therapeutic efficacy of inhaled corticosteroids is mediated via the inhibition of NF-κΒ-regulated gene expression [5]. NF-κΒ is a transcription factor that regulates several cytokine and adhesion molecule genes expressed during allergic inflammation, including colony-stimulating factor, TNF-α, intercellular adhesion molecule (ICAM)-1, and vascular cell adhesionmolecule-1 (VCAM-1) [5]. Dexamethasone (Dex) is a synthetic glucocorticoid that exerts anti-inflammatory effects by inhibiting the NF-κΒ signaling pathway [6]. However, the molecular mechanism of Dex in pulmonary inflammatory diseases remains unclear.

Long non-coding RNAs (lncRNAs) are involved in several biological processes, including cell growth, cell differentiation, and the cell cycle as well as in the progression and metastasis of cancer [7, 8]. Researchers have suggested that inflammatory cytokines, particularly NF-κΒ, play critical roles in regulating lncRNAs in human diseases [9]. miR-489 targets CHRF to inhibit MyD88 and Smad expression in pulmonary fibrosis [10]. In addition, in patients with COPD, differentially expressed NEAT1 correlated with increased inflammation [11]. Protein phosphatase ε (PTPRE) is involved in accelerating pulmonary allergic inflammation [12]. Therefore, we hypothesized that specific lncRNAs regulate the pathological processes underlying pulmonary inflammation.

In this study, we targeted specific lncRNAs that have been reported to be correlated with the inflammatory response in order to characterize their mechanistic roles in lung inflammatory diseases. The inflammatory response was induced via IL-1β and LPS using previously described methods in A549 cells [13–15] and was then attenuated using Dex. We quantified the changes in lncRNA expression during IL-1β and Dex treatment in A549 cells. In addition, we determined the functional roles of candidate lncRNAs in regulating the inflammatory response under Dex treatment in a pulmonary inflammation cell model. Because the functions of lncRNAs are usually performed by targeting miRNAs, we identified the miRNA targets of lncRNAs as well as quantified their expression levels to characterize their functions.

## Materials and methods

### Cell culture and treatment

A549 were obtained from Shanghai Cell Bank (Shanghai, China) and were cultured at 37 °C with 5% CO_2_. Cells were maintained on high-glucose DMEM supplemented with 10% fetal bovine serum (Gibco, USA). Cells were cultured, amplified, and passaged. After 3 days, cells were digested and pelleted via centrifugation. Cells morphology was observed using a light microscope and suspended at a concentration of 1 × 10^6^/mL. For constructing the inflammatory cell model, 10 ng/mL IL-1β (Peprotech, USA) or 1 μg/mL LPS (Sigma, USA) was added and incubated for 8, 16, and 24 h. To determine the effect of Dex (Sigma), different concentration of Dex (10 nM, 100 nM, 1000 nM, and 10,000 nM) and 10 ng/mL IL-1β were added at the same time and the cells were incubated for 24 h. To assess the functions of lncH19 and miR-324-3p, 10 ng/mL IL-1β and 1 μM Dex were simultaneously added and the cells and supernatants were harvested for further studies.

### MTS assay

Cells were suspended at a density of 1 × 10^6^/mL and were seeded into 96-well plates with 100 μL per well. Then, 10 ng/mL IL-1β was added to the cells with or without Dex (1 μM) for 24 h following transfection with or without an miRNA inhibitor for 24 h. Cells were examined at 1, 2, and 3 days. MTS was added, and after 3 h of incubation, the optical density of the cells was detected at 490 nm.

### Cell apoptosis assay

Cells at a density of 5 × 10^5^ cells/well were seeded into 6-well plates overnight at 37 °C. After treatment, cells were then pelleted and washed with PBS. The cells were resuspended in 1× binding buffer, followed by the addition of 5 μL of 7-AAD staining solution and 5 μL of APC-conjugated Annexin V. Samples were tested using the FACS Calibur flow cytometer. The percentage of apoptotic cells was determined.

### Lentivirus preparation and infection

The lncH19 sequence was obtained from NCBI (no. NR_002196.2). The antisense sequence of lncH19 (5′- CGGCAAGAAGCGGGTCTGTTTCTTT-3′) was synthesized and cloned into the LV3 vector together with the inverted repeat sequence. The empty vector LV3 was used as a control. The lentivirus solution was prepared by Shanghai Majorbio (China). A549 cells were seeded into 96-well plates at a density of 3 × 10^4^ cells/well. The virus solution was diluted in a 10× gradient at five different concentrations with DMEM. The supernatant with the culture medium was discarded from each well and supplemented with 100 μL of the virus solution, with different concentrations in each well. Saline solution, instead of a virus solution, was used as a control. Cells were incubated at 37 °C with 5% CO_2_ for 24 h. The cell culture supernatant was replaced with 100 μL of freshly prepared DMEM. Cells were then incubated for 72 h. RT-PCR was performed to detect lncH19 expression. miRNA inhibitor and the negative control were transfected using lipofectamine 3000 as per the manufacturer’s instructions (Thermo Fisher, USA).

### ELISA to detect the levels of inflammatory cytokines

The levels of cytokines in the cell culture supernatant were determined using ELISA kits for TNF-α, IL-6, and IL-8 (Cusabio, Wuhan, China). Briefly, 96-well plates were precoated with TNF-α-, IL-6-, and IL-8-specific human antibodies. Human TNF-α, IL-6, and IL-8 were used to prepare the standard titration curve. Samples and standards were added into 96-well plates and incubated for 120 min at 37 °C. Then, biotin-labeled antibody was added and the plates were incubated for 60 min at 37 °C. After washing the plates three times, HRP–avidin was added and the plates were incubated for 60 min at 37 °C. Following three washes, TMB substrate was added and plates were incubated for 25 min at 37 °C and in the dark. A stop solution was added, and the optical density of the samples was measured at 450 nm via photospectrometry.

### RT-PCR

The expression levels of the mRNAs of inflammatory genes, including IKβ-α, IKKβ, IL-6, IL-8 and TNF-α, lncRNAs, and miRNAs were verified via RT-PCR. The M-MLV Reverse Transcriptase (Promega, USA) was used to synthesize cDNA. PCR reactions were prepared using the GoTaq qPCR Master Mix (Promega, USA) and were performed on the ABI 7500 system (Applied Biosystem, USA). The PCR program was as follows: 95 °C for 30 s, followed by 40 cycles at 95 °C for 5 s and then at 60 °C for 30 s. The housekeeping genes GAPDH and U6 were used to normalize the expression levels. The primer sequences are shown in Table 1.

**Table 1:** Primers of mRNA, lncRNA, and miRNA

Name	sequence(5′-3′)
H-IL-6-F	ACTCACCTCTTCAGAACGAATTG
H-IL-6-R	CCATCTTTGGAAGGTTCAGGTTG
H-IL-8-F	GACCACACTGCGCCAACAC
H-IL-8-R	CTTCTCCACAACCCTCTGCAC
TNF-α-F	CTGCACTTTGGAGTGATCGG
TNF-α-R	GCTTGAGGGTTTGCTACAACAT
H-Iκκβ-F	CGATGGCACAATCAGGAAACAGGT
H-Iκκβ-R	ATTGGGGTGGGTCAGCCTTCTC
H-IKB-α-F	ACACCTTGCCTGTGAGCAGG
H-IKB-α-R	AGCACCCAAGGACACCAAAA
β-Actin-F	CATGTACGTTGCTATCCAGGC
β-Actin-R	CTCCTTAATGTCACGCACGAT
H-lncMALAT1-F	AAAGTCCGCCATTTTGCCAC
H-lncMALAT1-R	ACAACTCGCATCACCGGAAT
H-lncHotair-F	GCAGTGGGGAACTCTGACTC
H-lncHotair-R	TTGAGAGCACCTCCGGGATA
H-lncH19-F	GACATCTGGAGTCTGGCAGG
H-lncH19-R	CTGCCACGTCCTGTAACCAA
H-lncNeat1-F	GGCAGGTCTAGTTTGGGCAT
H-lncNeat1-R	CCTCATCCCTCCCAGTACCA
hsa-mir-346-RT	GTCGTATCCAGTGCAGGGTCCGAGGTATTCGCACTGGATACGACCCGCTC
hsa-mir-346-F	GGTCTCTGTGTTGGGCGTCT
hsa-miR-18a-3p-RT	GTCGTATCCAGTGCAGGGTCCGAGGTATTCGCACTGGATACGACCCAGAA
hsa-miR-18a-3p-F	ACTGCCCTAAGTGCTCCTT
hsa-mir-324-3p-RT	GTCGTATCCAGTGCAGGGTCCGAGGTATTCGCACTGGATACGACCCAGCA
hsa-mir-324-3p -F	CCCACTGCCCCAGGTGCT
hsa-miR-146b-5p-RT	GTCGTATCCAGTGCAGGGTCCGAGGTATTCGCACTGGATACGACACCAGA
hsa-miR-146b-5p-F	GCCCTGTGGACTCAGTT
hsa-miR-18b-5p-RT	GTCGTATCCAGTGCAGGGTCCGAGGTATTCGCACTGGATACGACCTAACT
hsa-miR-18b-5p-F	GCGTAAGGTGCATCTAGTGCAG
hsa-mir-19a-RT	GTCGTATCCAGTGCAGGGTCCGAGGTATTCGCACTGGATACGACTCAGTT
hsa-mir-19a-F	TGTGCAAATCTATGCAAA
hsa-miR-19b-3p-RT	GTCGTATCCAGTGCAGGGTCCGAGGTATTCGCACTGGATACGACTCAGTT
hsa-miR-19b-3p-F	GTGTGCAAATCCATGCAA
Universe-R	GTGCAGGGTCCGAGGT
U6-F	CGCTTCGGCAGCACATATAC
U6-R	CGAATTTGCGTGTCATCCTTG

### Western blotting

Cells were lysed in 1% SDS lysis buffer. The BCA assay was used to determine protein concentrations. Proteins were separated using 10% SDS–PAGE gels. Proteins were then transferred onto polyvinylidene fluoride membranes. Nonfat milk in PBS was used to block the membrane at room temperature for 1 h. The membrane was incubated overnight at 4 °C with the primary antibody [p-P65 (ab76302; Abcam), P65 (8242; CST), ICAM-1 (sc-107; Santa Cruz), VCAM-1 (sc-13,160; Santa Cruz), and GAPDH (HC301; Transgen)]. After several washes with PBS, the membranes were incubated with the blocking buffer and secondary antibody coupled to horseradish peroxidase for 2 h at room temperature. The complexes formed on the membrane were then detected using ECLplus (Amersham Biosciences/GE Healthcare, Velizy, France).

### Statistical analysis

The data are presented as the mean ± standard deviation (SD), *N* = 3. The non-parametric t-test and Kruskal–Wallis test were used to analyze differences between two groups and those among multiple groups, respectively. *P* < 0.05 was considered to indicate a statistically significant difference. The IBM SPSS statistics software 22.0 (SPSS Inc., Chicago, IL, USA) was used for statistical analysis.

## Results

### Dex attenuates the inflammatory response

**Fig. 1:**
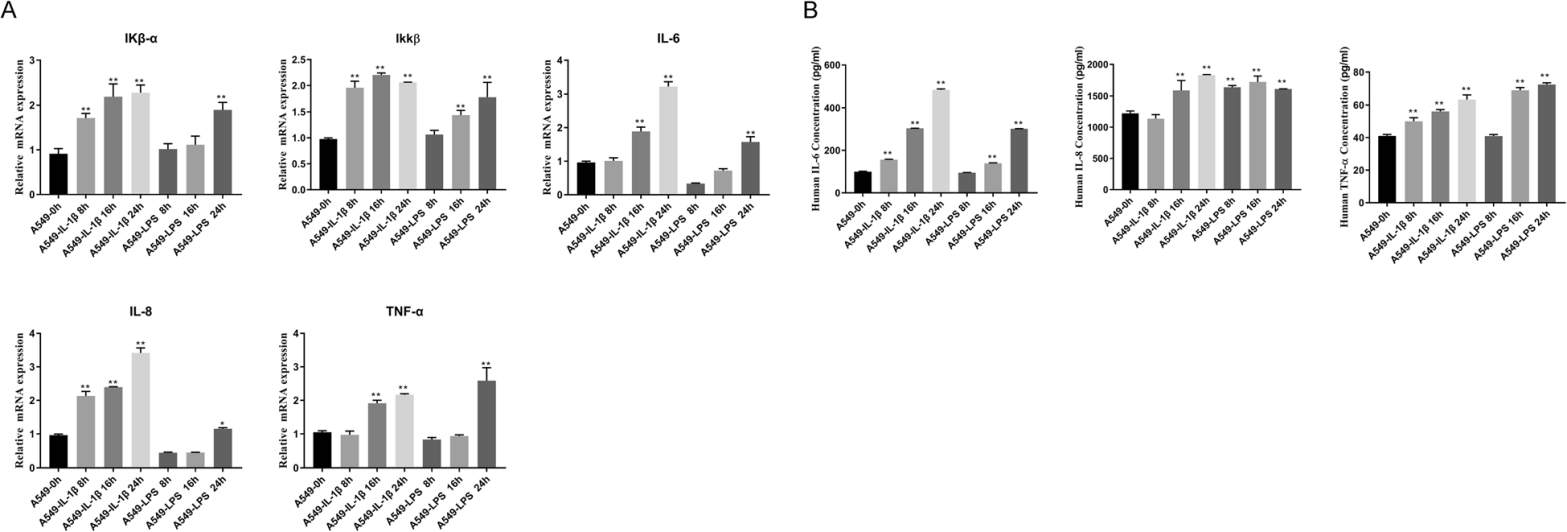
**a** pulmonary inflammatory cell model was established by inducing A549 cells with IL-1β (10 ng/mL) or LPS (1 μg/mL) for different time periods. IL-1β proved to be a more robust inflammatory cell model. A. RT-PCR determination of the corresponding mRNA levels of the inflammatory genes of IKβ-α, IKKβ, IL-6, IL-8, and TNF-α; GAPDH was used as the housekeeping gene. **b**. Levels of the inflammatory cytokines IL-6, IL-8, and TNF-α were determined via ELISA. Data are presented as mean ± SD *N* = 3. **P < 0.01 vs. A549 0 h. IL-1β: interleukin-1β, LPS: lipopolysaccharide

To investigate the effects of Dex, we constructed a pulmonary inflammatory cell model using IL-1β and LPS. A549 cells were used for the assay and were treated with 10 ng/mL IL-1β or 1 μg/mL LPS in DMEM. The cells incubated with the reagents were collected after 8, 16, and 24 h, and the total RNA was isolated. RT-PCR was conducted to confirm the mRNA expression levels of IKβ-α, IKKβ, IL-6, IL-8, and TNF-α. Generally, the results showed that IL-1β performed better against LPS because the mRNA levels of most of the inflammatory cytokines, except for IL-6 and TNF-α, were significantly upregulated at 8 h; in addition, the mRNA levels of all other cytokines were two- to four-fold greater at 24 h (Fig. 1a). The secretary cytokines in the cell culture medium, including IL-6, IL-8, and TNF-α were determined via ELISA. The results were consistent with that of RT-PCR and showed that IL-1β was generally more effective and that an effect can be observed at 8 h (Fig. 1b). Therefore, we decided to use IL-1β with 24-h incubation to construct the pulmonary inflammatory cell model.

**Fig. 2:**
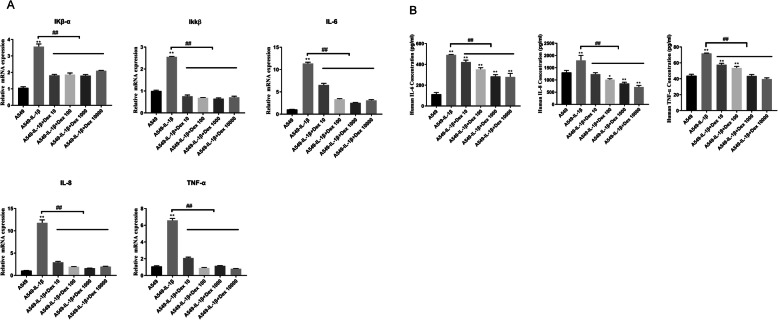
Different concentrations of Dex (10 nM, 100 nM, 1000 nM, and 10,000 nM) can attenuate the inflammatory response induced by IL-1β (10 ng/mL). IL-1β and Dex were added at the same time as the stimuli. **a**. mRNA levels of the inflammatory genes were determined via RT-PCR. **b**. Levels of the inflammatory cytokines were determined via ELISA. Data are presented as mean ± SD. *N* = 3. *P < 0.05 vs. A549 0 h, **P < 0.01 vs. A549 0 h, ##*P* < 0.01. Dex: dexamethasone, IL-1β: interleukin-1β

Following the same principle, we used the cell model to characterize the effects of Dex treatment. We incubated A549 cells with IL-1β and Dex at different concentrations, ranging from 10 nM to 10,000 nM, for 24 h. As mentioned above, RT-PCR and ELISA were used to evaluate the effects of Dex. The mRNA expression levels of IKβ-α, IKKβ, IL-6, IL-8, and TNF-α, significantly increased following IL-1β treatment (*P* < 0.05). After adding Dex, the mRNA levels decreased to their baseline levels, indicating that Dex supplementation can attenuate the inflammatory response (Fig. 2a). With respect to the levels of secretary inflammatory cytokines, compared with the control group, the levels of IL-6, IL-8, and TNF-α significantly increased following IL-1β treatment. However, when cells were supplemented with Dex, the levels of cytokines decreased; both 1 and 10 μM Dex significantly decreased the levels (Fig. 2b). Taken together, these observations suggest that Dex plays a role in attenuating the inflammatory response.

### Verification of the lncRNAs involved in the attenuation of the inflammatory response

**Fig. 3:**
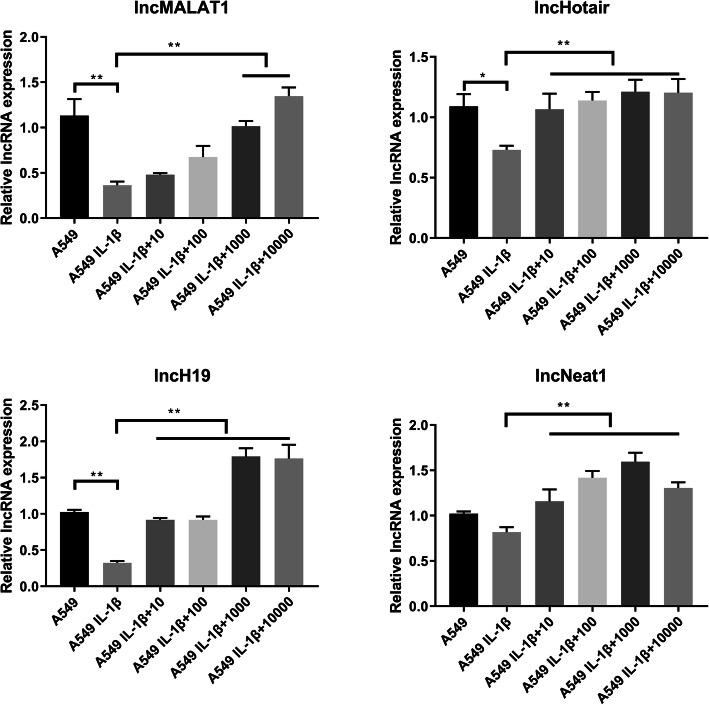
lncMALAT1, lncHotair, lncH19, and lncNeat1 were downregulated when A549 was treated with IL-1β (10 ng/mL) but were upregulated when the inflammatory response was attenuated following the application of Dex (1 μM); IL-1β and Dex were added at the same time as the stimuli. Changes in lncH19 were evident. Data are presented as mean ± SD. *N* = 3. *P < 0.05, **P < 0.01. Dex: dexamethasone, IL-1β: interleukin-1β

Based on our preliminary findings, we targeted four lncRNAs, namely lncMALAT1, lncHotair, lncH19, and lncNeat1, to determine which of these lncRNAs are involved in processes underlying inflammation attenuation [16–20]. RT-PCR was conducted to determine lncRNA levels following treatment with IL-1β and Dex. The results showed that the four candidate lncRNAs were downregulated following IL-1β treatment; however, lncRNA levels increased after Dex supplementation (Fig. 3). In general, lncRNA levels were most clearly observed after treatment with 1 and 10 μM Dex (*P* < 0.05). Among the lncRNAs, the level of lncH19 exhibited the highest increase, approximately two-fold, following treatment with 1 and 10 μM Dex. Therefore, lncH19 and 1 μM Dex were selected for further functional validation.

**Fig. 4:**
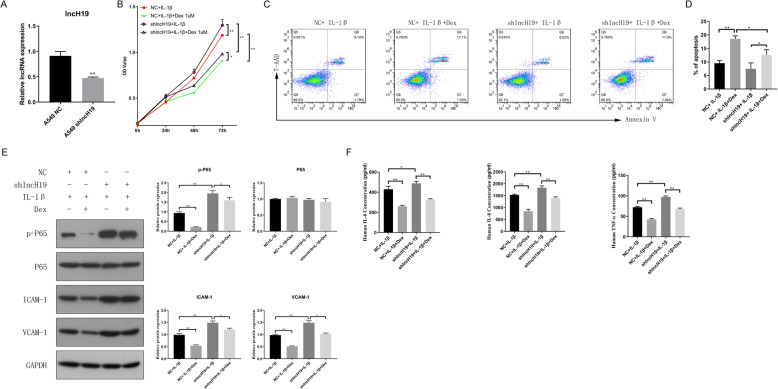
When lncH19 was inhibited but cells were treated with IL-1β (10 ng/mL) with or without Dex (1 μM) at the same time, cell proliferation increased, cell apoptosis decreased, and the protein levels of inflammatory genes increased, promoting the phosphorylation of P65, ICAM-1, VCAM-1, and inflammatory cytokines. **a**. lncH19 expression was reduced, indicating that an lncH19-inhibited cell line was generated. **b**. Cell proliferation of A549 NC and A549 shlncH19 cells treated with IL-1β with or without Dex. **c**. Representative cell apoptosis diagram, as measured via flow cytometry. Upper left is the fragment and damaged cells, upper right is the late apoptosis and dead cells, lower left is the normal cells of negative control, and lower right is the early apoptotic cells. The total percent of apoptosis cells was calculated by the sum of cells in the upper right and lower right. **d**. Percentage of apoptotic cells. **e**. Western blotting for determining the protein levels of inflammatory genes. **f**. ELISA for assessing the levels of inflammatory cytokines. Data are presented as mean ± SD. *N* = 3. *P < 0.05, **P < 0.01. Dex: dexamethasone, IL-1β: interleukin-1β

To verify the functions of lncH19, the corresponding shRNA was designed and infected into A549 cells using lentivirus. The performance of the specific shRNA was assessed via RT-PCR. LncH19 expression showed a 50% decrease after transfection with the specific shRNA (Fig. 4a). The cells were then treated with IL-1β and Dex (1 μM) to investigate the effect of decreased lncH19 expression. Cell proliferation was assessed via the MTS assay. Treatment with 1 μM Dex clearly inhibited cell proliferation, which might only be a side effect. When lncH19 expression was inhibited, cell proliferation increased relative to the group of normal expression of lncH19 (Fig. 4b). We then further validated the functions of lncH19 via the cell apoptosis assay using flow cytometry. Compared with the percentage of apoptotic cells without Dex treatment, apoptotic cells increased approximately two-fold when Dex was supplemented. Cell apoptosis was inhibited and reduced by 30–50% when lncH19 was inhibited under IL-1β and Dex treatment compared with normal expression of lncH19 under IL-1β and Dex treatment (Fig. 4 c & d 4). Western blotting was conducted to determine the protein level of the genes that might be involved in the inflammatory response, including P65, p-P65, ICAM-1, and VCAM-1. The protein level of P65 did not change. For the rest of the genes, Dex supplementation clearly decreased protein levels; however, lncH19 inhibition could increase protein levels (Fig. 4e). Therefore, given the consistency of these results with those obtained via flow cytometry and the MTS assay, the proteins p-P65, ICAM-1, and VCAM-1 are likely involved in Dex regulation. The cell culture supernatant was then collected and the levels of the inflammatory cytokines, including IL-6, IL-8, and TNF-α, were determined. Cytokines decreased in concentration by approximately 50% following Dex addition. However, lncH19 inhibition increased the levels of inflammatory cytokines (Fig. 4f). Therefore, Dex can attenuate the inflammatory response, and lncH19 plays an important role in the inflammatory response.

### Validation of the candidate miRNAs regulated by lncH19

**Fig. 5:**
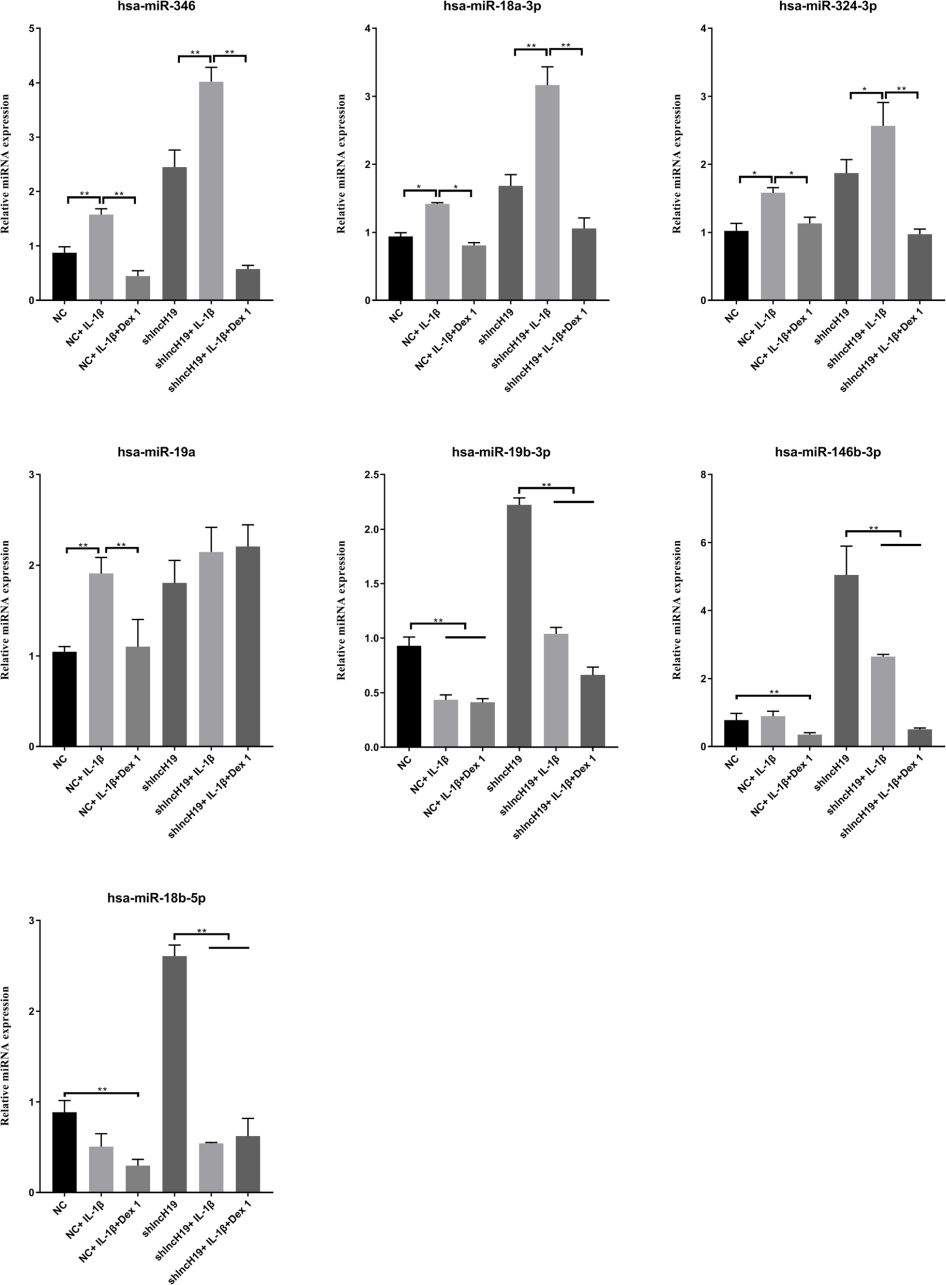
RT-PCR was conducted to determine the levels of miRNAs predicted to be targets of lncH19 with or without treatment with IL-1β (10 ng/mL) or IL-1β (10 ng/mL) and Dex (1 μM) added at the same time; trends in the expression of miR-346, miR-18a-3p, and miR-324-3p were consistent in A549 NC and A549 shlncH19. Data are presented as mean ± SD. *N* = 3. **P < 0.01. Dex: dexamethasone, IL-1β: interleukin-1 β

**Fig. 6:**
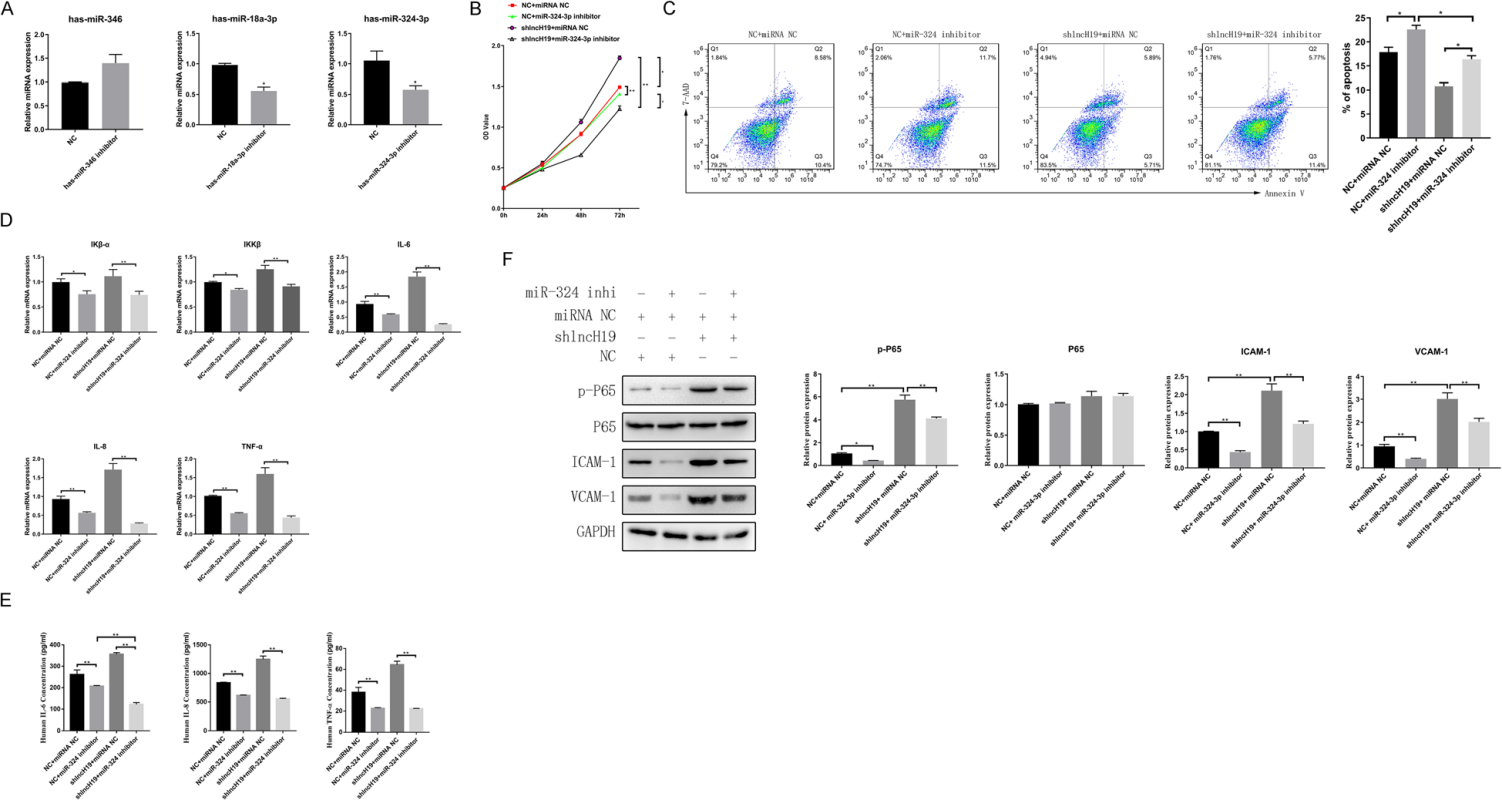
The attenuation of lncH19 by miR-324-3p was inhibited as the inflammatory response attenuated under treatment with IL-1β (10 ng/mL) and Dex (1 μM) at the same time. **a**. Levels of the candidate miRNAs, as determined via RT-PCR, for examining the performance of the miRNA inhibitor. **b**. Cell proliferation was determined using the MTS assay. **c**. Cell apoptosis was determined via flow cytometry. **d**. mRNA levels of the inflammatory cytokines. **e**. ELISA for detecting the levels of the inflammatory cytokines. **f**. Western blot for detecting the levels of the inflammatory genes. Data are presented as mean ± SD. N = 3. *P < 0.05, **P < 0.01. Dex: dexamethasone. IL-1β: interleukin-1 β; NC + miRNA NC: shNC + IL-1β + Dex + miRNA NC; NC + miR-324-3p inhibitor: shNC + IL-1β + Dex + miR-324-3p inhibitor; shlncH19 + miRNA NC: shlncH19 + IL-1β + Dex + miRNA NC; shlncH19 + miR-324-3p inhibitor: shlncH19 + IL-1β + Dex + miR-324-3p inhibitor

Miranda v3.3a was used to predict the target miRNAs of lncH19. The miRNAs are listed in Table 2. Because lncH19 expression was upregulated following Dex addition and because the upregulation of lncRNA tends to result in the downregulation of miRNAs, we searched for downregulated miRNAs following Dex addition. We observed that miR-346, miR-18a-3p, and miR-324-3p were downregulated; therefore, they were chosen for further validation (Fig. 5). The corresponding miRNA inhibitors were designed and transfected into A549 cells. The performance of the inhibitors was assessed via RT-PCR. For both miR-18a-3p and miR-324-3p, the corresponding inhibitors clearly decreased the expression of miRNAs by approximately 50%. The inhibitor of miR-324-3p was significantly more effective compared to that of miR-18a-3p (Fig. 6a). Therefore, we further investigated the functions of miR-324-3p and divided the cells into four groups: shNC + IL-1β + Dex + miRNA NC (NC + miRNA NC), shNC + IL-1β + Dex + miR-324-3p inhibitor (NC + miR-324-3p inhibitor), shlncH19 + IL-1β + Dex + miRNA NC (shlncH19 + miRNA NC), and shlncH19 + IL-1β + Dex + miR-324-3p inhibitor (shlncH19 + miR-324-3p inhibitor). The MTS assay was conducted to assess cell proliferation. Compared with the NC + miR-324-3p inhibitor group, the cell proliferation of the NC + miRNA NC group significantly increased at 72 h (*P* < 0.05). Cell proliferation of the shlncH19 + miRNA NC group increased from an OD value of 1.5 to 2.0 compared with in the NC + miRNA NC group. Cell proliferation was lower in the shlncH19 + miR-324-3p inhibitor group than in the shlncH19 + miRNA NC group (Fig. 6b). Therefore, these results suggest that lncH19 expression inhibits cell proliferation and miR-324-3p expression promotes cell proliferation. We also conducted the cell apoptosis assays using flow cytometry to determine the functions of miR-324-3p. lncH19 inhibition reduced the number of apoptotic cells by ~ 30%; however, miR-324-3p inhibition clearly enhanced cell apoptosis compared with in the NC + miRNA NC group (Fig. 6c). Compared with the NC + miRNA NC group, the mRNA levels of IKβ-α, IKKβ, IL-6, IL-8, and TNF-α were upregulated when lncH19 was inhibited but downregulated when miR-324-3p was simultaneously inhibited (Fig. 6d). The cell culture supernatant was collected to determine the levels of the cytokines IL-6, IL-8, and TNF-α. The levels of the cytokines significantly increased when lncH19 was inhibited by shRNA relative to the NC + miRNA NC group. However, the levels of cytokines significantly decreased when the expression of miR-324-3p was simultaneously inhibited, indicating that the inflammatory response was suppressed (Fig. 6e). Western blotting was performed to determine the protein levels of inflammatory genes, including p-P65, P65, ICAM-1, and VCAM-1. There was no significant change in the protein level of P65. lncH19 inhibition increased the protein levels of p-P65, ICAM-1, and VCAM-1 relative to the NC + miRNA NC group. However, protein levels were decreased when miR-324-3p was downregulated by an inhibitor simultaneously. These findings suggest that miR-324-3p is involved in lncH19 regulation. lncH19 might target and inhibit miR-324-3p expression and thus regulate inflammation.

**Table 2:** Prediction of the target miRNAs of lncH19

lncRNA	miRNA	numBindingSitesPredicted	numTargetsPer100bp	specificBindingSitesPredicted
NR_002196.2	hsa-miR-346	5	0.22	252;435;579;887;1114
NR_002196.2	hsa-miR-324-3p	5	0.22	569;580;723;831;1103
NR_002196.2	hsa-miR-18a-3p	3	0.13	577;674;828
NR_002196.2	hsa-miR-18b-5p	2	0.09	137;1667
NR_002196.2	hsa-miR-146b-3p	2	0.09	257;746
NR_002196.2	hsa-miR-19b-3p	1	0.04	2023
NR_002196.2	hsa-miR-19a-3p	1	0.04	2034

## Discussion

In this study, IL-1β-induced inflammatory A549 cells were attenuated by Dex to aid the study of lncRNAs. Initially, we targeted lncMALAT1, lncHotair, lncH19, and lncNeat1 and evaluated their expression levels. The results showed that lncH19 was most closely related to the symptoms of inflammation because its expression decreased following IL-1β addition to cells but increased following Dex supplementation. In addition, lncH19 expression negatively correlated with miR-324-3p. We showed that Dex attenuates the inflammatory response by targeting a cascade involving lncH19 and miR-324-3p.

Pulmonary inflammatory diseases, such as asthma, are diseases associated with increase inflammation and loss of bronchial epithelial cells. Increased levels of inflammatory cytokines and cell apoptosis are one of the characteristics of bronchial epithelial cells in patients of pulmonary inflammatory diseases [16–18]. Therefore, drugs with the ability to suppress inflammation and benefit the survival of epithelial cells can improve the outcomes of patients with pulmonary inflammatory disease. In the present study, we used IL-1β-induced A549 cells to mimic inflammatory epithelial cells. Based on this inflammatory epithelial cell model, we reveled that lncH19 is suppressed upon IL-1β induction and is partially restored by Dex, suggesting its role in inflammatory epithelial cells.

LncH19 was discovered in the 11p15.5, H19/IGF2 locus [19]. It is highly expressed in the fetus but begins to be silenced after birth, with its expression restricted to a few tissues, such as the mammary gland, uterus, and adrenal gland [20]. Existing evidence suggests that H19 mutation in mouse zygotes results in prenatal lethality. This indicates that H19 plays an important role in cell growth and development [21]. Therefore, our findings are consistent with those of previous studies that lncH19 can regulate cell proliferation. In addition, we suggested that lncH19 is involved in modulating apoptosis in inflammatory epithelial cells. Notably, lncH19 is associated with the NF-κB pathway and is thus involved in the regulation of cell growth in patients with multiple myeloma [9]. NF-κB is an important family of transcription factors involved in cell differentiation, apoptosis, and immunity. Activation of the NF-κB pathway is involved in most cellular processes in cancer transformation, including the inhibition of cell differentiation and cell apoptosis, promotion of cell proliferation, angiogenesis, and potential metastasis [22]. At present, evidence shows that NF-κB plays a role as a pro-inflammatory mediator in the production of cytokines, chemokines, and cell adhesion molecules in inflammatory cells and structural cells, such as lung epithelial cells [23]. However, its relationship with lncH19 remains unclear in the context of lung inflammatory epithelial cells. In the present study, we showed that induction of the inflammatory response by IL-1β reduced lncH19 expression, whereas Dex supplementation, which can attenuate the inflammatory response, significantly increased lncH19 expression. The upregulation of lncH19 expression correlates with the attenuation of the inflammatory response in lung epithelial cells. Taken together, these findings indicate that lncH19 is involved in the anti-inflammatory processes associated with pulmonary inflammatory disease. However, because lncRNAs usually inhibit miRNA targets, lncH19 might not directly target NF-κB. Therefore, we suspect that lncH19 targets and inhibits NF-κB and possibly other anti-inflammatory factors.

Further studies on lncH19 revealed that the expressions of miR-346, miR-17a-3p, and miR-324-3p were inhibited by lncH19 overexpression. Following the detection of cell proliferation and cell apoptosis, miR-324-3p inhibition revealed a highly significant correlation between cell proliferation and apoptosis. Therefore, we suspect that lncH19 regulates the inflammatory response by regulating miR-324-3p. miR-324-3p is a commonly expressed miRNA that regulates the processes underlying cancer. The main target of miR-324-3p is SMAD7. According to Xu et al., the miR-324-3p/SMAD7 axis plays a significant role in regulating cell growth and apoptosis in patients with nasopharyngeal cancer [24]. The overexpression of SMAD7 is associated with the development of skin, pancreatic, lung, and colon cancer in the processes of cell growth and apoptosis [25]. Previous studies have shown that SMAD7 is associated with the NF-κB pathway and therefore regulates the cell cycle in cancers [26]. Inflammation often changes the expressions of p65, ICAM-1, and VCAM-1; therefore, their expression levels can be used as indicators of the inflammatory response. Previous studies have also shown that p65 is associated with NF-κB activation [5]. By characterizing the expression of cytokines, we have confirmed that both lncH19 and miR-324-3p could be important factors involved in regulating the inflammatory response. Therefore, regulation of the inflammatory response might be achieved via the regulation of the SMAD7/NF-κB pathway via a regulatory network between lncH19 and miR-324-3p in which lncH19 targets miR-324-3p. However, this proposed regulatory cascade requires further validation in the future.

Our results showed that Dex can attenuate the inflammatory response in IL-1β-induced inflammatory cells. The lncH19/miR-324-3p axis might play an important role in regulating the inflammatory response, and thus, regulate cell proliferation and cell apoptosis. Future studies should examine additional genes targets of the lncH19/miR-324-3p axis, including SMAD7 and NF-κB, to further elucidate the regulatory processes associated with the lncH19/miR-324-3p axis. Furthermore, the expression of lncH19/miR-324-3p should be quantified in animal models and patients with pulmonary inflammatory disease before and after the use of corticosteroids. The anti-inflammatory effect of Dex in asthma is regulated by the lncH19/miR324-3p pathways in animal model. The correlation between changes in lncH19 and miR-324-3p expression in specific pulmonary inflammatory diseases, such as asthma, needs to be studied in the future because our hypothesized mechanism is based on an IL-1β-induced epithelial cell model. In addition, we can also search for possible ncRNAs using the RNA-seq technology because lncRNAs may also interact with other ncRNAs.

## Conclusion

In summary, we demonstrated for the first time that Dex can attenuate the inflammatory response in inflammatory epithelial cells, an important pathological change closely associated with pulmonary inflammatory diseases, such as asthma, by regulating the lncH19/miR-324-3p cascade.

## Data Availability

All data generated or analyzed during this study are included in this published article.
